# Sexually transmitted infections based on the syndromic approach in Gondar town, northwest Ethiopia: a retrospective study

**DOI:** 10.1186/1471-2458-13-143

**Published:** 2013-02-16

**Authors:** Beyene Moges, Gizachew Yismaw, Afework Kassu, Berihun Megabiaw, Shitaye Alemu, Bemnet Amare, Dagnachew Muluye

**Affiliations:** 1 Department of Immunology and Molecular Biology, School of Biomedical and Laboratory Sciences, College of Medicine and Health Sciences, University of Gondar, PO Box 196, Gondar, Ethiopia; 2 Department of Medical Microbiology, School of Biomedical and Laboratory Sciences, College of Medicine and Health Sciences, University of Gondar, PO Box 196, Gondar, Ethiopia; 3 Department of Epidemiology and Biostatistics, Institute of Public Health, College of Medicine and Health Sciences, University of Gondar, PO Box 196, Gondar, Ethiopia; 4 Department of Internal Medicine, College of Medicine and Health Sciences, University of Gondar, PO Box 196, Gondar, Ethiopia; 5 Department of Biochemistry, College of Medicine and Health Sciences, University of Gondar, Box 196, Gondar, Ethiopia

**Keywords:** Sexually transmitted infections, Syndromic approach, Ethiopia

## Abstract

**Background:**

Sexually transmitted infections are among the most common causes of illnesses in the world and have far reaching health, social and economic consequences. They are important because of their magnitude, potential complications and interactions with HIV/AIDS. Though the problem may be generally similar to other developing countries, there is scarce information on the incidence and prevalence of sexually transmitted infections in Ethiopia. This study was then aimed to determine the magnitude of sexually transmitted infections among patients visiting a clinic in Gondar town, Northwest Ethiopia.

**Methods:**

Medical records of patients who visited the clinic from January 2011 to December 2011 were reviewed. Sociodemographic and clinical data were extracted using data extraction form. The data were entered and analyzed using SPSS version 16 statistical package. Descriptive statistics and Chi-square tests were carried out.

**Results:**

A total of 1071 clients visited the clinic during the study period. Among these, 383 (35.8%) had complained symptoms of sexually transmitted infections. The mean (SD) age of the patients was 26.8 ± 7.4 years. The commonest chief complaints were vaginal discharge (38.4%) and urethral discharge (13.6%). Seventy seven percent of the cases did not bring their sexual partners for treatment.

**Conclusion:**

There was a high magnitude of STIs in the clinic according to the syndromic approach. However, the actual prevalence of STIs and the associated factors in the community need to be determined through further studies. The results of this study also urge the need for evaluation of the syndromic approach and test for antimicrobial resistance.

## Background

It is estimated that more than 340 million new cases of curable sexually transmitted infections (STIs) occur every year throughout the world among adults aged 15–49 years, with the second largest proportion in the region of sub Saharan Africa [1]. Excluding HIV, STIs account for a substantial proportion of outpatient health care visits among adults of 15–49 years and in most nations STIs rank among the top five leading causes that individuals seek health care [2]. In fact, reported STIs represent only the “tip of the iceberg” because most infections—typically more than half of any specific diagnosis regardless of bacterial or viral etiology are entirely asymptomatic or if symptoms exist often unrecognized [3-5]. This is especially true for women [6-8].

STIs are important because of their magnitude, potential complications and their interaction with HIV/AIDS. The World Health Organization estimated that each year more than 340 million new curable STIs occur in reproductive-aged men and women; this excludes the estimated 33 million new cases of HIV as well as estimated 100 million plus infections caused by other viral STIs each year [9]. Disproportionately, it affects the health and social wellbeing of women by producing significant impact on their reproductive potential. There is little information on the incidence and prevalence of STIs in Ethiopia. There are many reasons for this, but the major contributing factor is that most people with STIs do not seek treatment at public health facilities as they will have minor or no symptoms. They usually tend to take self-prescribed drugs or go to private pharmacies to buy treatment without consulting trained health workers [10].

In Ethiopia, relatively few epidemiological surveys had been carried out on the prevalence and incidence of STIs. However, the problem of STIs in Ethiopia is generally believed to be similar to other developing countries. The Integrated Disease Surveillance Team of the Ministry of Health compiled 58,623 and 27,947 STI cases in 2002 and 2003 respectively using routine quarterly reports [11].

In an STI survey conducted in Ethiopian two third (65.9%) presented with vaginal discharge, a quarter (25.1%) with urethral discharge, and 76 (17.6%) with genital ulcer. *N. gonorrheae* was the leading pathogen that caused urethral discharge in males as compared to females with vaginal discharge and bacterial vaginosis was the common cause of vaginal discharge in females. Syphilis (24.0%) was the second leading cause of genital ulcer in males as compared to females [12]. As to our knowledge, there are no studies conducted to show the magnitude of STIs in Northwest Ethiopia. Therefore, the objective of this study was to determine the magnitude of STIs among patients visiting a clinic in Gondar town, northwest Ethiopia.

## Methods

A cross-sectional study was conducted at a clinic in Gondar town from January 2011 to December 2011. The clinic is serving a population of more than 300,000 people living in the town. This health care facility lacks the equipment and trained personnel required for etiological diagnosis of STIs. Thus, the clinic uses the syndromic approach to diagnose and treat STIs. The syndromic management approach is based on the identification of consistent groups of symptoms and easily recognized signs (syndromes), and the provision of treatment that will deal with the majority of or the most serious organisms responsible for producing a syndrome [13]. This included counseling and detailed sexual history taking, taking sociodemographic characteristics, and detailed clinical signs and symptoms with elaboration of the chief complaints. Physical examination was performed systematically and meticulously. Speculum examination was also part of the physical examination in women. Proctoscopy was not available in the clinic; hence, it wasn’t done. In summary, flow chart was used during the initial evaluation of patients in the clinic. The clinic also used the etiologic approach at times to diagnose gonorrhea and syphilis using gram staining and syphilicheck rapid tests, respectively. All the 383 patients who visited the clinic with STIs symptoms during the study period were included. Medical records of these patients were reviewed by investigators and both socio-demographic and clinical data were extracted using data extraction form.

### Data analysis

The data was double entered and analyzed using SPSS version 16 statistical software package. Descriptive statistics and Chi-square test were carried out.

### Ethical considerations

The study was conducted after ethical approval from the Institutional Review Board of the University of Gondar. No patient identifiers were used and all the information was collected confidentially.

## Results

### Socio-demographic characteristics

A total of 1071 clients visited the clinic during the study period. Among this, 383 (35.8%) had complained of one or more of the STIs symptoms. Most cases were aged between 15 and 49 years (97.9%). The mean (SD) age of the clients was 26.8 ± 7.4SD years. The mean age of females and males was 25.6 ± 6.4 and 31.2 ± 8.9, respectively. Females constituted the majority, 301 (78.6%) of STI cases. About 91% of the cases were from the town while the rest 8.6% of the patients were from the surrounding rural areas.

### Clinical profile of study subjects

The most frequent chief complaints were vaginal discharge (38.38%), combined symptoms (29%) and urethral discharge (13.58%). Burning sensation during urination (9.14%), vulvar vulvar itching (3.92%), LAP (2.87%), genital ulcer (2.09%) and genital swelling (1.04%) were the other chief complaints reported by the patients (Table 1). Seven of the 58 (12.07%) urethral discharge cases were bacteriologically diagnosed to be due to *N. gonorreae and* four out of the eight (50%) genital ulcer cases were serologically diagnosed to be syphilis.

**Table 1:** Clinical profile of study subjects at Gondar town clinic, northwest Ethiopia

**Characteristics**		**Frequency**	**%**
**Diagnosis**	STI	365	95.3
	Gonorrhea	7	1.8
	Syphilis	4	1.1
	PID	7	1.8
**Chief complaints**	Vaginal discharge	147	38.4
	Urethral discharge	52	13.6
	Genital ulcer	8	2.1
	Vulvar itching	15	3.9
	Burning sensation during urination	35	9.1
	LAP	11	2.9
	Swollen genitalia	4	1.1
	Combined symptoms	111	29
**Partner treatment**	Yes	88	23.0
	No	295	77.0
**Re-treatment**	Yes	8	2.1
	No	375	97.9

LAP = Lower Abdominal Pain, PID = Pelvic Inflammatory Disease.

As per the national guideline [13] vaginal discharge/vulvar itching was treated with a combination of Ciprofloxacin 500 mg po stat, Doxycyclin 100 mg po bid for 7 days, and Metronidazole 500 mg po bid for seven days while urethral discharge/burning sensation during urination was treated with a combination of Ciprofloxacin 500 mg po stat and Doxycyclin 100 mg po bid for 7 days. Genital ulcer was treated with Bezanthine penicillin 2.4 million units IM stat or with a combination of Doxycyclin 100 mg po bid for 14 days and Ciprofloxacin 500 mg po for three days. About 1% (4/383) of cases were not given any kind of antibiotic treatment. The reasons for not treating were not specified.

Two hundred ninety five (77%) of the cases did not bring their sexual partners for treatment. More males (53.7%) tend to bring their sexual partners than females (14.6%) which is significantly associated with p-value of <0.001 (χ^2^ = 55.5). Most (71.2%) of the cases who brought their sexual partners for treatment were between 15 and 29 years of age which is significantly associated with p-value of 0.002 (χ^2^ = 14.9). Among those who brought their sexual partner for treatment, 13.6% were from the rural areas.

## Discussion

STIs are among the leading public health problems that individuals seek health care in the town. Our finding is in agreement to the report by Dallabetta et al. where STIs, in most nations, are ranked among the top five leading causes that individuals seek health care [2]. Similarly, even in developed countries like the United States, five of the 15 most commonly reported notifiable diseases are STIs (gonorrhea, Chlamydia, HIV, syphilis and hepatitis B) including the first and second most commonly reported diseases, gonorrhea and Chlamydia, respectively [14]. In fact, reported STIs represent only the “tip of the iceberg” because most infections—typically more than half of any specific diagnosis regardless of bacterial or viral etiology—are entirely asymptomatic and/or unrecognized [3-5]. This is especially true for women [6-8]. Furthermore, adequate data are not available in developing countries to even analyze reporting rates in those settings [2]. This obviates that the actual situation in the study area could even be harsher as only symptomatic cases came to the clinic. There could also be unreported symptomatic cases due to stigma and discrimination, fear of potential conflict with sexual partner especially in the married group, self prescription of medicines from pharmacies, preference to traditional healers, and because of the general poor health seeking behavior of the community. There could be a hell of asymptomatic cases, subclinical cases, and cases with minor symptoms. There could also be unnoticed symptomatic cases. The level of awareness to distinguish between abnormal vaginal discharges from the normal ones, in women, could be one factor. The fact that STI was more reported by women in this study could also be one other sign of high prevalence of STIs in the community as women tend to be more asymptomatic or subclinical in most of the cases [6-8]. However, the actual prevalence of STIs and the aforementioned assumptions need to be proved through further studies.

In line with the 2006 WHO report the age distribution showed patients in the reproductive age group (15–49 years) were the major STI affected (97.9%) portion of the population [1]. Our result is in agreement with the report by Klouman et al. in Tanzania [15] where they found the highest rate of STIs among 25–34 years of age females and 35 to 44 age group of males Considerably STIs affected women (78.6%) in the town. This could be due the geographic location of the clinic and increased level of awareness to STI treatment in towns than rural areas. As discussed earlier, factors affecting the health seeking behavior of individuals in the community need to be explored.

In this study, the most frequent chief complaints of study participants were vaginal discharge (38.38%), combination of the sign and symptoms (28.7%) and urethral discharge (13.58%). The reason for higher proportion of vaginal discharge could be due to the fact that majority of the participants were females. The majority of STI causing organisms be it fungal, bacterial and/or protozoal has also manifestations of vaginal discharge in women. Higher rates of similar STI syndromes were also reported in by Wolday et al. Addis Ababa, Ethiopia [12]. Seven out of 58 (12.07%) urethral discharge cases were bacteriologically diagnosed to be due to *N. gonorreae.* Four out of the eight (50%) genital ulcer cases were serologically diagnosed to be syphilis. As the etiologic approach was used sporadically and randomly in the study clinic, the actual proportion of etiologic cases for discharge or ulcerative STIs needs to be determined. This could strengthen the empirical syndromic approach by showing the frequent etiologic agent in the community.

Treatments of the cases were delivered as per the national guideline of syndromic management of sexually transmitted infections [13]. Despite having its own huge contribution for drug resistance, the syndromic management might be helpful for the existing relief of patients. Re-treatment was reported by 2.7% (8/301) of female cases but not males. Among the cases visited the clinic for re-treatment, 62.5% were with chief complaint of vaginal discharge. This might indicate that the syndromic flowchart for the management of vaginal discharge does not work well for controlling sexually transmitted infections in women because this symptom is a poor proxy for endocervical Chlamydia and gonorrhea [16-18]. On the other hand, treatment failure due to several other factors could be the reason for the re-treatment like failure to bring sexual partner, antimicrobial resistance, poor compliance to treatment, HIV co-infection (which we were unable to retrieve from the participants’ record) and another infection. This obviates an urgent need for the evaluation of the sensitivity and specificity of the syndromic approach and test for antimicrobial resistance. In addition to this, some cases might visit a different health institution for the re-treatment or may get self prescribed medication from pharmacies with a resultant under estimation of re-treatment.

In the case of this study where 77% of the cases didn’t bring their sexual partners for treatment, the percentage of cases visiting clinics for re-treatment is even underestimated. On top of this, the new cases in the study clinic might have previous history of treatment in other clinics in the town. Besides, cases may not return to the same clinic for re-treatment for several reasons. Among those who brought their sexual partner for treatment, 13.6% were from the rural areas. Age and sex were found to be significantly associated with partner treatment. This shows the need to fostered counseling of patients while they are getting the service. Higher proportion of older age groups brought their sexual partners for treatment compared to other age groups. Young adults may not be transparent for their partners and prohibit discussions for such issues, due to cultural and other influences, which is again an area where an intervention should have to be taken.

Our study is limited to a clinic where only health care seeking individuals are found. Many important variables were not also included in the study due to incomplete/absence of the variables in the record. Population based studies could be helpful to show the real picture of STIs in the area.

## Conclusion

There was a high magnitude of STIs in the clinic. However, the actual prevalence of STIs and the associated factors need to be determined through further studies. The results of this study also urge the need for evaluation of the syndromic approach and test for antimicrobial resistance.

## Abbreviations

AIDS: Acquired Immune Deficiency Syndrome; HIV: Human Immunodeficiency Virus; LAP: Lower Abdominal Pain; PID: Pelvic Inflammatory Disease; STI: Sexually Transmitted Infections; SD: Standard Deviation; SPSS: Statistical Packages for Social Sciences.

## Competing interests

The authors declare that they have no competing interests.

## Authors’ contributions

BM and DM were involved in the study conception and design, data analysis, and drafting the manuscript. BA, BM, GY, SA and AK were involved in data analysis, drafting the manuscript and reviewing the manuscript. All the authors have read, edited and approved the manuscript.

## Pre-publication history

The pre-publication history for this paper can be accessed here:

http://www.biomedcentral.com/1471-2458/13/143/prepub
